# Recent trends in vanadium-based SCR catalysts for NOx reduction in industrial applications: stationary sources

**DOI:** 10.1186/s40580-022-00341-7

**Published:** 2022-11-19

**Authors:** Bora Ye, Bora Jeong, Myeung-jin Lee, Tae Hyeong Kim, Sam-Sik Park, Jaeil Jung, Seunghyun Lee, Hong-Dae Kim

**Affiliations:** 1 Green Materials & Processes R&D Group, Korea Institute of Industrial Technology, Ulsan, 44413 Republic of Korea; 2 Department of Chemical and Molecular Engineering, Hanyang University ERICA, Ansan, 15588 Republic of Korea; 3 Center for Bionano Intelligence Education and Research, Hanyang University ERICA, Ansan, 15588 Republic of Korea; 4 R&D Center, NANO. Co., Ltd, Sangju, 37257 Republic of Korea

**Keywords:** Vanadium-based catalysts, Stationary sources, Selective catalytic reduction, NOx removal efficiency, Catalyst poisoning

## Abstract

Vanadium-based catalysts have been used for several decades in ammonia-based selective catalytic reduction (NH_3_-SCR) processes for reducing NO_*x*_ emissions from various stationary sources (power plants, chemical plants, incinerators, steel mills, etc.) and mobile sources (large ships, automobiles, etc.). Vanadium-based catalysts containing various vanadium species have a high NO_*x*_ reduction efficiency at temperatures of 350–400 °C, even if the vanadium species are added in small amounts. However, the strengthening of NO_*x*_ emission regulations has necessitated the development of catalysts with higher NO_*x*_ reduction efficiencies. Furthermore, there are several different requirements for the catalysts depending on the target industry and application. In general, the composition of SCR catalyst is determined by the components of the fuel and flue gas for a particular application. It is necessary to optimize the catalyst with regard to the reaction temperature, thermal and chemical durability, shape, and other relevant factors. This review comprehensively analyzes the properties that are required for SCR catalysts in different industries and the development strategies of high-performance and low-temperature vanadium-based catalysts. To analyze the recent research trends, the catalysts employed in power plants, incinerators, as well as cement and steel industries, that emit the highest amount of nitrogen oxides, are presented in detail along with their limitations. The recent developments in catalyst composition, structure, dispersion, and side reaction suppression technology to develop a high-efficiency catalyst are also summarized. As the composition of the vanadium-based catalyst depends mostly on the usage in stationary sources, various promoters and supports that improve the catalyst activity and suppress side reactions, along with the studies on the oxidation state of vanadium, are presented. Furthermore, the research trends related to the nano-dispersion of catalytically active materials using various supports, and controlling the side reactions using the structure of shaped catalysts are summarized. The review concludes with a discussion of the development direction and future prospects for high-efficiency SCR catalysts in different industrial fields.

## Introduction

Fine particulate matter has been designated as a Class 1 carcinogen by the World Health Organization and is classified into two types according to its emission source. The particulate matter that comes out as a solid from the source is called primary particulate matter. Secondary particulate matter is generated through a chemical reaction in which sulfur oxides and nitrogen oxides, which are representative gaseous pollutants emitted from industrial sites, combine with water vapor, ozone, or ammonia in the atmosphere [1]. Nitrogen oxide (NOx), a representative air pollutant that acts as a major precursor to particulate matter, is emitted at high concentrations and is mainly generated from fixed sources, such as manufacturing, power plants, industrial boiler, gas turbines, production processes, as well as mobile sources, such as ships and automobiles [2]. The NOx itself is not only harmful to the human body but also causes various environmental problems, such as acid rain, global warming, and smog. The NOx is highly mobile as it is readily carried over long distances by the wind, causing worldwide problems [3].

Methods to reduce the NOx content are classified into pre-combustion control, combustion control, and post-combustion control methods [4]. The pre-combustion control method involves reducing the nitrogen content by refining the nitrogen component in the fuel. Combustion control technology reduces the NOx emission by controlling the temperature, oxygen content, and residence time of gas during the combustion process. Combustion control technologies include low NOx burner and flue gas recirculation. Post-combustion control involves the removal of NOx through an additional after-treatment system; selective catalytic reduction (SCR) and selective non-catalytic reduction (SNCR) are the most widely used technologies for the post-combustion control [4].

However, pre-combustion and combustion control do not remove NOx with sufficient efficiency to comply with the increasingly stringent emission regulations. Therefore, post-combustion control technology is widely used in industry [5].

**Fig. 1:**
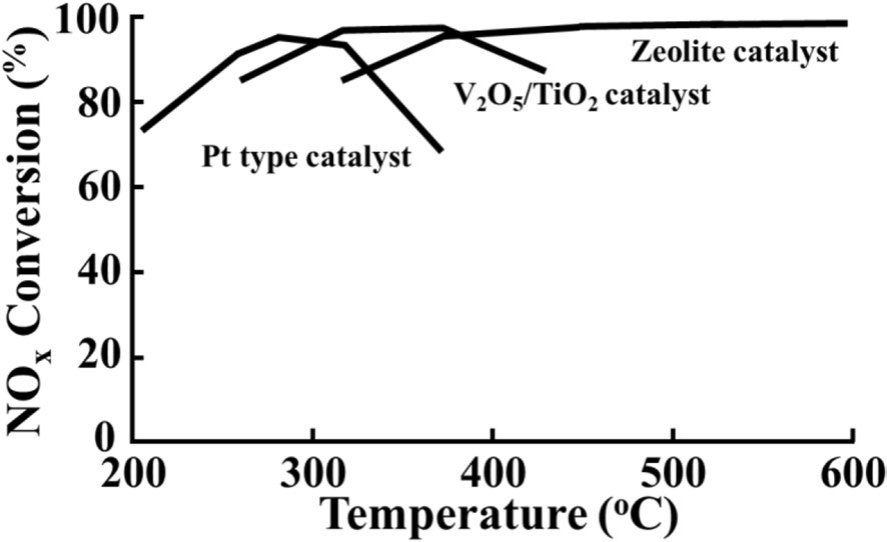
Three major families of SCR catalyst [2]

The SNCR and SCR technologies are the most typical processes used for post-combustion control. In SCR process, combustion gas containing NOx and a reducing agent (such as NH_3_ or urea) passes through a catalytic layer to selectively reduce NOx to nitrogen and water vapor. In a SNCR process, the reagent converts NOx into nitrogen and water vapor through reactions without catalysts. Because a catalyst is not used, a reducing agent is injected at high temperature (900–1100 °C) to achieve the activation energy required for the reaction [6]. Therefore, SCR technology is widely used as a NOx reduction process to comply with the emission regulations. Several types of SCR catalysts are used including, metal oxide-based, zeolite-based, alkaline-earth metal-based, and rare-earth-based catalysts. Among these, the V–W-based catalysts are the most widely used, as they exhibit high NOx removal efficiency of more than 90% at temperatures over 380 °C [2, 7]. Zeolite-based catalysts are also used in some catalyst applications because they have a high specific surface area and a wide operating temperature range, but they have the disadvantage of exhibiting high activity only when pretreatment is performed in a moisture-free condition [7]. The characteristics of representative SCR catalysts are described in Fig. 1 and Table 1.

**Table 1:** Operating characteristics of different SCR catalysts [2]

Medium temperature VNX™ catalyst (V_2_O_5_/TiO_2_)	High temperature –ZNX™ catalysts (zeolite)	Low temperature – LT catalyst (Pt-based)
260–425 °C most broadly used 10–15 years of experience sulfur tolerant	345–590 °C very high NOx conversion very low NH_3_ slip NH_3_ destruction sulfur tolerant above 425 °C	150–300 °C narrow temperature window temperature window shifts not sulfur tolerant

The SCR reactions are usually defined as either “standard SCR” or “fast SCR.” In general, NOx is composed of 95% NO and 5% NO_2_; therefore, the reduction reaction is followed in the standard condition. However, in the case of fast SCR reaction, in which NO, NO_2_, and NH_3_ react in the absence of oxygen, the reaction is completed more rapidly due to the rapid oxidation [5]. Sometimes, an oxidizing device such as a diesel oxidation catalyst (DOC) is connected to the front of the catalyst to oxidize NO to NO_2_. The catalytic activity in the low-temperature region is enhanced through fast SCR, which is faster than the existing standard reaction. The fast SCR reaction generally shows excellent reactivity when NO and NO_2_ are present in a 1:1 molar ratio [5].

Oxygen presence condition

1 $${\text{4NH}}_{{3}} + {\text{ 4NO }} + {\text{ O}}_{{2}} \to {\text{4N}}_{{2}} + {\text{ 6H}}_{{2}} {\text{O }}:{\text{ Standard SCR}}$$

2 $${\text{4NH}}_{{3}} + {\text{ 2NO}}_{{2}} + {\text{ O}}_{{2}} \to {\text{3N}}_{{2}} + {\text{ 6H}}_{{2}} {\text{O}}$$

Oxygen absence condition

3 $${\text{2NO }} + {\text{ 2NO}}_{{2}} + {\text{ 4NH}}_{{3}} \to {\text{4N}}_{{2}} + {\text{ 6H}}_{{2}} {\text{O }}:{\text{ Fast SCR}}$$

4 $${\text{4NH}}_{{3}} + {\text{ 6NO}} \to {\text{5N}}_{{2}} + {\text{ 6H}}_{{2}} {\text{O}}$$

Side reactions include ammonia oxidation at high temperatures, oxidation of SO_2_ caused by excess vanadium content, and formation of ammonium salts by the reaction of unreacted ammonia and SO_3_. The formation of ammonium sulfate corrodes the post-treatment facilities, leading to reduced catalytic activation due to the blocked surface of the catalyst [8].

The SCR catalysts are divided into the three types: honeycomb, plate, and corrugated. Industrial V_2_O_5_–WO_3_/TiO_2_ catalysts (usually containing 0.5–3 wt% V_2_O_5_ and 5–10 wt% WO_3_) exhibit high de-NOx efficiency, and excellent resistance to sulfur (SO_2_) and H_2_O. Nevertheless, they also exhibit several disadvantages, such as high and narrow effective temperature range (300–400 °C), and a tendency to oxidize SO_2_ to SO_3_. The SO_3_ reacts with NH_3_ to form ammonium sulfate or bisulfate, shortening the lifetime of the catalyst [9]. As a result, in major industrial applications such as thermoelectric power plants and vessels with SCR catalyst, frequent shutdowns are necessary due to operational and equipment limitations. Therefore, SCR catalysts with high de-NOx efficiency at low operating temperatures are required for economic benefits and reduced energy consumption [10]. In addition, operating limitations of the SCR system cause a temporary rise in the system temperature. Currently, in the case of small and medium-sized power generation facilities and general boilers such as the heat recovery system generator boiler, most of the horizontal reactors have a relatively high impact due to high temperature. Therefore, there is a need to develop a SCR catalyst with high activity and durability against thermal shock at high temperatures. Thus, it is necessary to develop a wide temperature range SCR catalyst which is capable of operating at the conventional low temperatures (200–250 °C), exhibits enhanced catalytic properties such as high de-NOx efficiency along with low SOx conversion, and has a long lifetime.

**Fig. 2:**
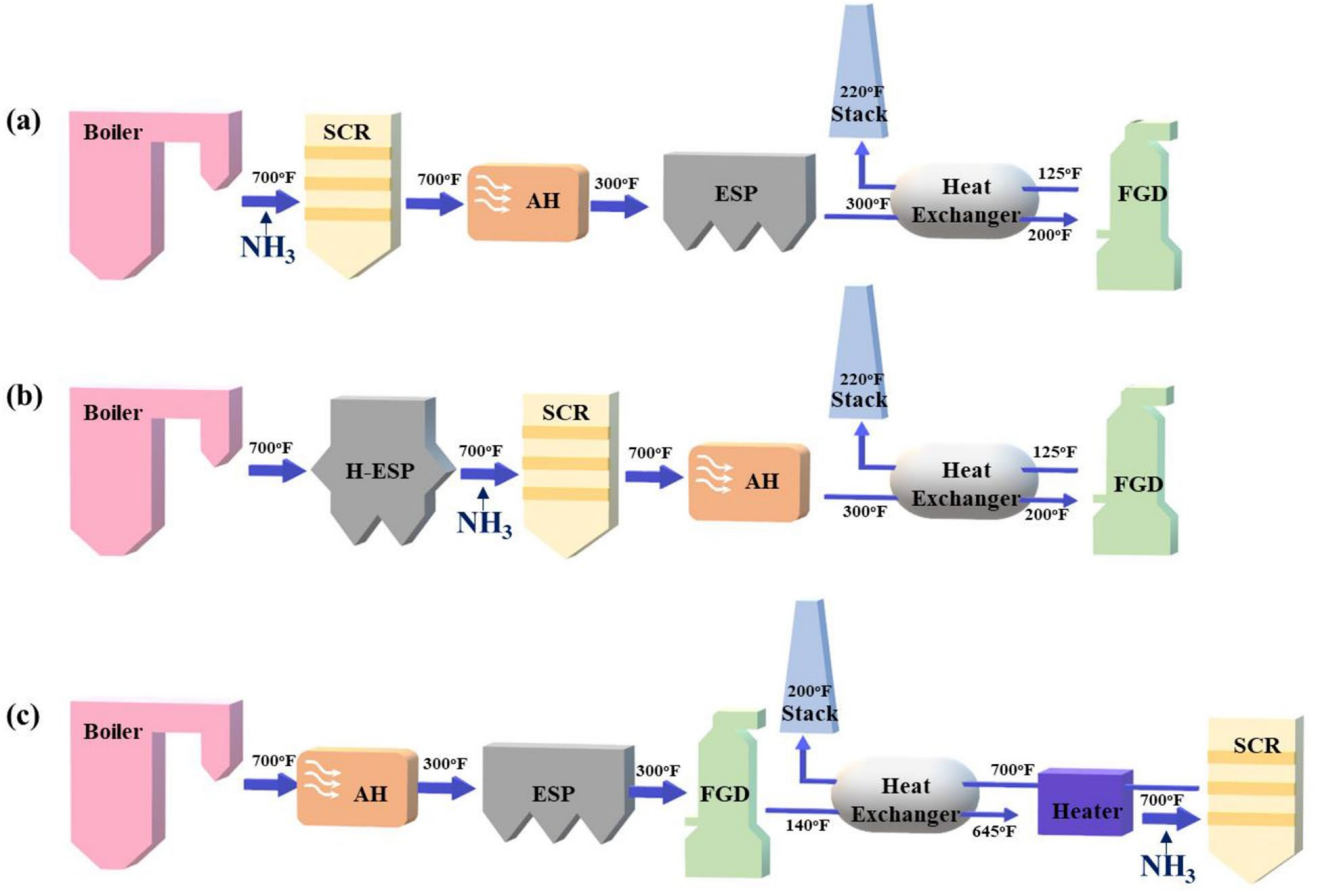
SCR configurations with typical system temperatures: **a** high-dust system, **b** low-dust system, and **c** tail-end system [11]

**Fig. 3:**
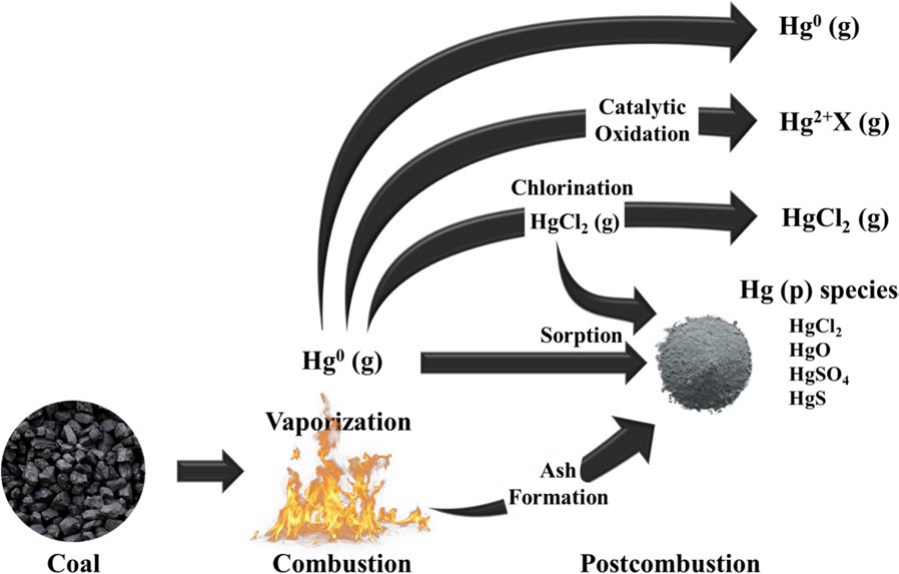
Potential mercury transformations during coal combustion and in the resulting flue gas [16]

**Fig. 4:**
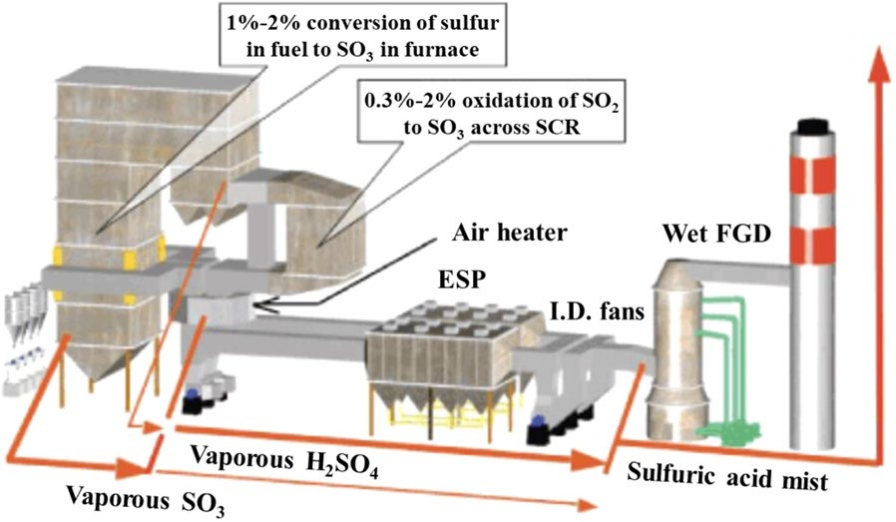
Oxidation of SO_2_ to SO_3_ in a boiler and SCR (Electric Power Research Institute) [18]. *ESP* electrostatic precipitator, *FGD* flue gas desulfurization system, *I.D* induced-draft, *SCR*  selective catalytic reduction system.

**Fig. 5:**
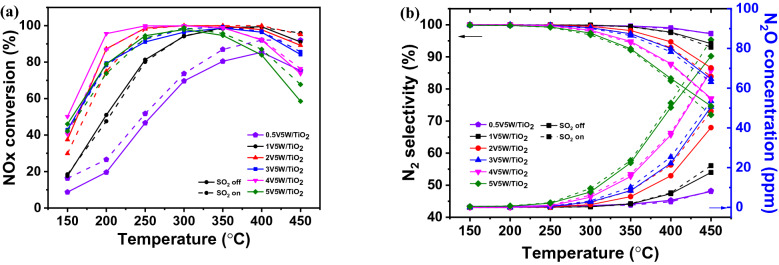
NOx removal efficiencies of the synthesized SCR catalysts according to vanadium content measured in the temperature range 150–450 °C **a** NOx conversion and **b** N_2_ selectivity and N_2_O concentration. Reaction conditions: [NOx] = 300 ppm, [NH_3_] = 300 ppm, [SO_2_] = 0 or 300 ppm, [O_2_] = 5%, balance N_2_, total flow 500 sccm, and the gas hourly space velocity (GHSV) = 60,000 h^−1^

## Applications of NH_3_-SCR catalysts for stationary sources

In stationary sources, various problems, such as the carbon content in fly ash, sulfur, alkali metals, the concentration of catalytic poisons in fuel, carbon monoxide (CO) emission, and corrosion may occur depending on the application [7]. The SCR facilities in stationary sources are generally classified into high dust configurations, low dust configurations, and tail-end systems, and are configured according to the application requirements (see Fig. 2) [11]. The variety of emission conditions from a stationary source require the NH_3_-SCR catalyst to operate under varied conditions and catalytic properties. Specifically, the catalysts must resist sulfur poisoning and minimize the oxidation of SO_2_ to SO_3_ because the SO_2_ concentration in the flue gas is relatively high [9].

Power plants generally use a high dust system, and, in this configuration, the SCR units are usually located directly after the boiler. Then an electrostatic precipitator and desulfurizer are installed in that order. This system has the advantage of having a high operating temperature without the need for additional heating devices, as the catalysts are installed at the end of the boiler. However, in facilities using biomass fuels, the catalytic activity decreases significantly due to physical poisoning (cell plugging of the monolith catalyst and wear of the plate catalyst) by alkali (earth) metals, and particulate matter in the exhaust gas significantly decreases the catalyst lifetime [12].

In a low dust configuration, the SCR reactor is located behind the hot-side electrostatic precipitator (H-ESP), reducing catalyst degradation by particulate erosion. However, this configuration requires the installation of an expensive H-ESP and a flue gas reheat system to maintain optimum operating temperatures [11].

Finally, in the tail end system, the catalytic system is placed downstream of the electrostatic precipitator and the desulfurization system. This system is mainly used in Western Europe, and because it removes both dust and SO_2_ from the catalyst, it is suitable for increasing the catalyst lifetime. However, in this system, NOx removal efficiency is drastically decreased because of the low-temperature regime it operates in. Although V-based catalysts are the most efficient in the temperature range of 300–400 °C, their efficiency rapidly decreases at lower temperatures, and the unreacted NH_3_ reacts with SO_2_ or H_2_O to form ammonia bisulfate. Therefore, additional flue gas reheating equipment is required for V-based catalysts used in this system. Reheating the system to the catalytic activation temperature sharply increases the maintenance cost of the SCR system. Therefore, it is necessary to study the potential of using low-temperature catalysts that exhibit activity at 200 °C or less for this configuration due to the problem of reheating the exhaust gas to the catalyst activation temperature [13]. Due to economic and spatial problems, SCR catalysts are currently positioned as tail-end systems. In particular, a temperature below 220 °C is required in incinerators as the SCR is located behind the baghouse filter [7].

Recently, problems related to the storage and transport of ammonia have led to suggestions that it be subject to environmental regulation in the United States, and emissions of ammonia slippage in the unreacted flue gas are stringently monitored in New Jersey and California. Several studies are underway to replace the reducing agent. Although hydrocarbon-reducing agents have attracted considerable attention as replacements for ammonia, their commercialization is difficult due to the requirements of high reaction temperatures and noble metal catalysts [7]. Studies related to “Enhanced SCR” show high NO reduction efficiency in the low-temperature range of ~ 200–350 °C with the addition of NH_4_NO_3_ along with NH_3_, a reducing agent. The resultant reaction is summarized in Eq. (5) [14].

5 $${\text{2NH}}_{{3}} + {\text{ 2NO }} + {\text{ NH}}_{{4}} {\text{NO}}_{{3}} \to {\text{3N}}_{{2}} + {\text{ 5H}}_{{2}} {\text{O}}$$

Variables that promote the enhanced SCR reaction at low temperatures include space velocity, reaction temperature, and injection amount of ammonium nitrate. When these parameters are optimized, a high NOx removal efficiency of 90% can be expected at a low temperature of 180 °C even with the use of a commercial V–W/TiO_2_ catalyst [14].

### Power plants

Power plants mainly use various fossil fuels, such as coal, heavy oil, and liquefied natural gas (LNG) to generate electricity. However, each fuel source poses a particular set of problems in the SCR plant; the deactivation mechanism of SCR catalysts used in coal-fired power plants is different from that of catalysts used in biomass power plants. In coal-fired power plants, catalyst deactivation by mercury and sulfate is the main factor. However, in biomass power generation, alkali (earth) metals act as the main cause of catalyst life reduction [15].

Coal fuel presents several problems. First, it is a representative anthropogenic source of mercury. Among heavy metals in coal, highly volatile mercury is converted into gaseous elemental mercury under high-temperature conditions (> 1400 °C) during combustion. The Hg present in flue gas exists in three main forms: Hg^0^ (elemental mercury), Hg^2+^ (oxidized mercury), and HgP (particulate mercury). First, Hg^0^(g), which is the gaseous elemental mercury present in the flue gas, reacts with other gaseous components and particulate matter and is converted into either HgP or oxidized mercury (Hg^2+^) [16]. In Fig. 3, HgP is collected in ESP facilities, and mercury oxide is removed with flue gas desulfurization (FGD) because it is soluble in water; however, gaseous elemental mercury is insoluble in water and is therefore difficult to reduce using the existing facilities.

**Fig. 6:**
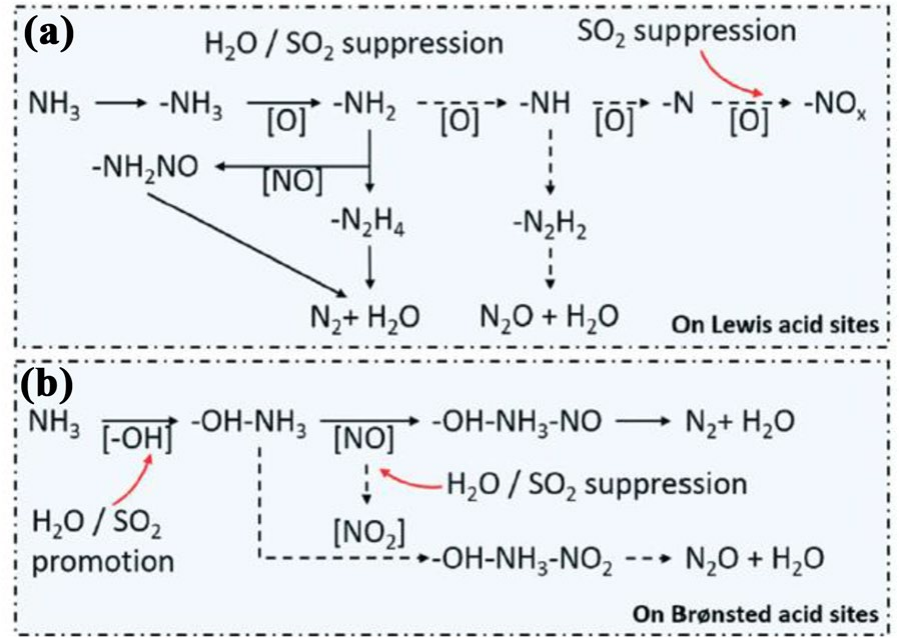
Proposed mechanism of NO reduction and N_2_O formation, as well as H_2_O/SO_2_ suppression effects, with the participation of **a** Lewis acid sites and **b** Brønsted acid sites over the Mn/Ti–Si catalyst [43]

To solve this problem, an SCR catalyst has been developed with elemental mercury oxidation capability, along with a separate oxidation catalyst. Although SCR technology is mainly used for NOx removal, many studies have shown that it can also be used to convert Hg^0^(g) into Hg^2+^. The oxidation of elemental mercury by the SCR catalyst is closely related to the chlorine content in the coal. As the chlorine content of coal increases, the oxidation of Hg^0^ to Hg^2+^ is promoted by the SCR catalyst, and as a result, the Hg^2+^ concentration at the end of the SCR process increases [16, 17].

Second, a problem is caused by the sulfur present in coal and heavy oil. During combustion, pyrite and organically-bound sulfur are mostly oxidized to SO_2,_ and a very small amount is converted to SO_3_ (SO_2_/SO_3_ ratio is generally ~ 40:1–80:1). Meanwhile, a portion (0.3–2%) of SO_2_ is oxidized to SO_3_ in the passage of the SCR facility (see Fig. 4) [18]. The SO_3_ in fuel forms various salts, such as H_2_SO_4_, NH_4_HSO_4_, and (NH_4_)_2_SO_4_ by reacting with H_2_O or NH_3_ in the exhaust gas, poisoning the catalyst and corroding the post-treatment facilities such as the air pre-heater and ESP [11].

The co-firing and burning of biomass fuel causes catalyst poisoning due to impurities in the fuel. The analysis of Danish cereal straw and wood chips, which are the most widely-used biomass fuels for power plants (Table 2), indicated the presence of 0.2–1.9 wt%. potassium, which is an alkali metal. Submicron aerosol particles in flue gases from straw combustion consist of almost pure potassium chloride and sulfate with small amounts of sodium, phosphorus, and calcium [19, 20].

**Table2:** Fuel data for Danish cereal straw and wood chips [20]

Chemical composition. Unit: wt% on dry basis	Straw		Wood chips	
	Typical	Variation	Typical	Variation
Ash	4.5	2–7	1.0	0.3–6
Volatiles	78	75–81	81	70–85
Hydrogen, H	5.9	5.4–6.4	5.8	5.2–6.1
Carbon, C	47.5	47–48	50	49–52
Nitrogen, N	0.7	0.3–1.5	0.3	0.1–0.7
Sulphur, S	0.15	0.1–0.2	0.05	< 0.1
Chlorine, Cl	0.4	0.1–1.1	0.02	< 0.1
Silicon, Si	0.8	0.1–1.5	0.1	< 1.1
Aluminum, Al	0.005	< 0.03	0.015	< 0.1
Iron, Fe	0.01	< 0.03	0.015	< 0.1
Calcium, Ca	0.4	0.2–0.5	0.2	0.1–0.9
Magnesium, Mg	0.07	0.04–0.13	0.04	< 0.1
Sodium, Na	0.05	< 0.3	0.015	< 0.1
Potassium, K	1.0	0.2–1.9	0.1	0.05–0
Phosphorus, P	0.08	0.03–0.2	0.02	< 0.1

Currently, as many countries increase the use of biomass for environmental reasons, the effect on the deactivation of the catalyst by the alkali metals K and Na and the alkaline earth metals Ca and Mg contained in biomass is attracting attention. The P_2_O_5_ reduces the pore volume of the catalyst surface and also reduces the catalytic activity. In addition, it reacts with H_2_O or O_2_ in the exhaust gas to form H_3_PO_4_. With an increase in the P_2_O_5_ concentration, the H_3_PO_4_ forms poly-, pyro-, tri-, and metaphosphoric acid chains, negatively affecting the catalyst activity [21]. Potassium (K) in fly ash generated during biomass combustion forms KCl and K_2_SO_4_ compounds, which are sticky particles with low melting points, and blocks the micropores of the catalyst to reduce its catalytic activity. Zheng et al. reported the need for additional research on catalyst poisoning caused by small amounts of phosphorus (P), calcium (Ca), and sodium (Na) [22]. In the research on catalyst deactivation by the formation of K compounds (e.g., KCl, K_2_SO_4_, and K_3_PO_4_) following the combustion of K-getter fuel, the polyphosphoric acid poisoning was relatively low compared to K poisoning. However, pore blocking and fouling occurred on the surface of the catalyst, which had a greater effect on deactivation than poisoning by K [22–24]. According to a study on the effect of the addition of inorganic substances to the SCR catalyst and poisoning, catalysts doped with alkali (earth) metals exhibit catalyst deactivation due to a reduction in the NH_3_ storage capacity, and a strong poisoning effect occurs in the order of K > Na > Ca > Mg [25].

Lastly, LNG-fired power generation is an eco-friendly power source to replace coal-fired power in Asia, and many new power plants are being built. As LNG is composed of ~ 72%–95% methane, ~ 3–13% ethane, ~ 1–4% propane, and ~ 1–18% nitrogen, it does not contain toxic substances such as sulfur and alkali (earth) metals, and there is almost no reduction in the catalyst life. However, as the main components are hydrocarbons, the process emits a large number of harmful substances, including carbon monoxide (CO) and unburned hydrocarbons (UHC).

As described above, various factors affect catalyst poisoning in power plants depending on the fuel used. Research on catalysts having anti-poisoning properties is an active field of study. In addition, a large amount of NOx is generated due to incomplete combustion during power plant startup, as the exhaust gas temperature does not reach the catalyst activation temperature and is discharged. Therefore, to control this problem, research on technology for storing NOx generated below the catalyst activation temperature by installing a NOx trap in front of the catalyst is also being conducted [26].

### Incinerators

Incinerators have a high dust configuration, and the SCR catalyst is placed at the rear end of the boiler; however, catalytic efficiency is severely reduced due to physical and chemical deactivation by fly ash present in the flue gas. Catalysts used in municipal waste incineration (MSWI) plants have reduced activity due to decreases in their specific surface area and pore sizes. One of the main causes is the change of the surface acid sites by alkali metals such as Na and K present in the flue gas [27, 28]. According to the findings of Jan et al., when domestic waste and sludge are incinerated simultaneously, the flue gas contains metals such as Ca, Si, Cl, S, K, Na, Pb, Zn, and P [27]. In particular, during co-incineration, a large amount of P is present in domestic sludge, and therefore, a larger amount of P is emitted than that formed during single combustion [29]. On the one hand, Castellino found that when the H_3_PO_4_ concentration reached 1000 ppm, the catalyst redox performance deteriorated, and the catalyst was deactivated after 24 h due to a decrease in the number of active vanadium species [23]. On the other hand, Cao et al. suggested that as P inhibits NH_3_ oxidation at temperatures above 300 °C, it prevents the formation of N_2_O and NOx, which are side reactants, and improves catalyst efficiency. To solve the problem of catalyst deactivation, the tail-end system is applied, and ESP and FGD are installed in front of the SCR to remove particulate matter, preventing catalyst poisoning. However, as the flue gas temperature rapidly decreases to approximately 160 °C, it is necessary to develop a low-temperature catalyst [30]. In addition, research on SCR catalysts for incinerators is mainly focused on the catalyst deactivation effect and reaction modeling for each element, along with studies on inhibiting the physicochemical poisoning of catalysts by fly ash and catalyst regeneration methods [27, 31].

### Cement, iron, and steel industry

The cement industry mainly uses the SNCR process to reduce NOx, but as air pollutant emission regulations are tightened around the world, hybrid SNCR–SCR and SCR technologies have attracted increased attention from the cement industry [32]. [32] While the amount of dust in the flue gas of a power plant is approximately 25 g/Nm^3^, the dust concentrations in the precalciner kiln system of a cement plant range from 50 to 100 g/Nm^3^. Therefore, the high dust configuration or semi dust application is mainly applied to the post-treatment facility. The semi dust application requires the development of a low-temperature catalyst because the temperature of the SCR facility decreases as dust is collected using a cyclone or ESP equipment in front of the SCR unit [33]. Therefore, there is an unfulfilled requirement for the development of catalysts for cement plants. These catalysts should be resistant to physicochemical poisoning by particulate matter such as fly ash, and exhibit high activity at low temperatures [34, 35].

The iron and steel process produces pollutants such as waste gas, water, and slag, the most serious of which is waste gas. Steelworks emit NOx from processes such as sintering, coking, and rolling, and a particularly large number of pollutants are emitted from the sintering process [36]. In the sintering process, limestone is mixed with iron ore in powder form and heated to process it into a sinter in the form of a homogeneous mass [37]. Steelworks generally use a high-dust configuration, and like the previous stationary sources, the demand for efficient low-temperature catalysts that can function below 240 °C is increasing. As SO_2_ is present in the exhaust gas, the poisoning of SCR catalysts by sulfur is a major problem [38]. In addition, even if a low-temperature catalyst is developed, the problem of catalyst poisoning due to moisture and sulfur remains. Therefore, research on sulfur- and moisture-resistant catalysts through the addition of a co-catalyst or by changing the catalyst support is an active field of investigation [39, 40]. According to Chu and Wang, there is considerable ongoing research related to the selection of various complex adsorbents to improve the efficiency of denitrification and desulfurization through the simultaneous removal of SO_2_ and NOx [41].

## Vanadium-based SCR catalysts

Vanadium, which is most widely used as a commercial catalyst, is considered a representative catalyst because it has high activity at ~ 350–400 °C even at ~ 1–2 wt% content of various vanadium chemical species such as monomeric, polymeric, and crystalline V_2_O_5_ [42]. Recently, the tail-end system has become more widely used, and the demand for low-temperature catalysts is increasing. However, active materials that can increase activity in the low-temperature region (< 200 °C), such as Mn and Ce, form salts such as MnSO_4_ or CeSO_4_ with the SO_2_ present in the exhaust gas. This results in the poisoning of the active catalyst sites, and the problem of activity deterioration continues to be an issue. Therefore, the catalytic activity temperature range is extended by increasing the vanadium content, making vanadium the major component in low-temperature catalysts. However, vanadium catalysts still have some problems. The operating temperature range is relatively narrow at ~ 350–400 °C, and at high temperatures, ammonia oxidation, SO_2_ oxidation, and N_2_O formation occur due to side reactions [5]. Because vanadium exhibits several problems, such as being easily sublimated and generating biological toxicity when used for a long time, it is necessary to minimize the vanadium content.

**Fig. 7:**
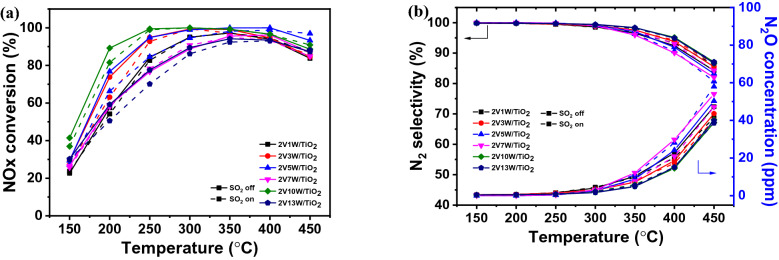
NOx removal efficiencies of the synthesized SCR catalysts according to tungsten content measured in the temperature range 150–450 °C **a** NOx conversion and **b** N_2_ selectivity and N_2_O concentration. Reaction conditions: [NOx] = 300 ppm, [NH_3_] = 300 ppm, [SO_2_] = 0 or 300 ppm, [O_2_] = 5%, balance N_2_, total flow 500 sccm, and the gas hourly space velocity (GHSV) = 60,000 h^−1^

An analysis of NOx conversion as a function of the vanadium content of a catalyst showed that when the vanadium content was between 0.5 and 1 wt%, the conversion efficiency was 19.57 and 51% at 200 °C, respectively. As the vanadium content increased from 2 to 5 wt%, it exhibited increased efficiency and an extended operating temperature range from 200 to 350 °C. When the temperature was greater than 400 °C, the efficiency decreased rapidly with vanadium content and was closely related to the amount of N_2_O generated. Figure 5 shows that as the vanadium content increases, the amount of N_2_O, generated due to one of the side reactions of the SCR reaction, increases rapidly, and N_2_ selectivity decreases. The main SCR side reactions by vanadium catalysts include N_2_O generation by NH_3_ oxidation and SO_3_ generation by SO_2_ oxidation. Extensive studies are required on vanadium content conditions and dispersibility improvements that inhibit these side reactions and yield high efficiency. Figure 7a shows the NOx conversion efficacy according to the presence of SO_2_ (off: solid line, on: dotted line); the condition without SO_2_ at low temperature (below 200 °C) shows high efficiency across the entire temperature range. There is no significant difference in the range of ~ 250–350 °C; however, the efficiency is increased when SO_2_ is added at a high temperature.

When SO_2_ is present at a low temperature, it is more readily adsorbed to the catalyst acid site than ammonia. It blocks the acid sites, which may cause a decrease in activity at low temperatures (Fig. 6) [43]. In addition, in the presence of SO_2,_ the amount of N_2_O generated may be reduced as the oxidation reaction of NH_3_ is suppressed. This is because SO_2_ is first adsorbed to the Brønsted acid site and Lewis acid site, and then the adsorption of NO is inhibited, leading to the preferential adsorption of NH_3_. The adsorbed NH_3_ improves the SCR reaction and suppresses side reactions.

**Fig. 8:**
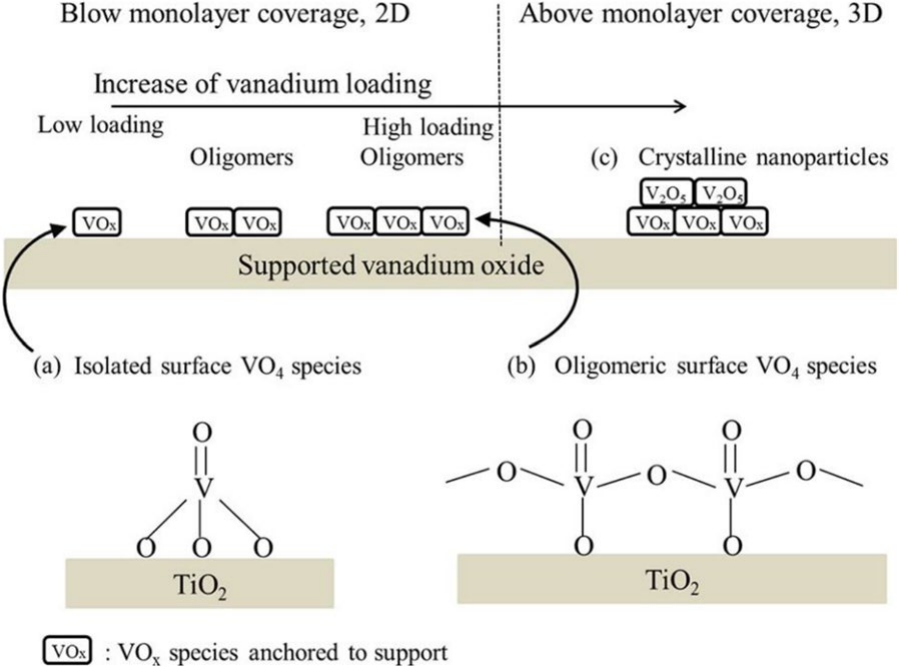
Structures of the dehydrated surface vanadate phases on TiO_2_:**a** isolated mono-oxo VO_4_, **b** oligomeric mono-oxo VO_4_, and **c** crystalline V_2_O_5_ nanoparticles on top of the surface vanadate monolayer [78]

As a commercial catalyst, tungsten or molybdenum are mainly used as co-catalysts for vanadium-based catalysts. Tungsten and molybdenum play a role in maintaining the structural and thermal stability of the catalyst and have the advantage of resistance to sulfur. Although the choice of tungsten or molybdenum depends on the intended use, molybdenum has the limitation of generating N_2_O at high temperatures.

Analyzing the catalyst properties according to tungsten content (Fig. 7) at a constant vanadium content shows that as the tungsten content increases, the NOx removal efficiency is improved at low temperatures. When 10 wt% of tungsten is added, the catalysts show the highest efficiency across the entire temperature range, but this decreases sharply at 13 wt% tungsten. The amount of N_2_O emitted showed almost no change according to the tungsten content. The value of the N_2_ selectivity calculated based on the amount of N_2_O generated showed an almost invariant range within ~ 80–87% at 450 °C. As the content of the co-catalyst WO_3_ was increased, the activity was improved at low temperatures. However, as it increases to 13 wt%, the dispersibility is lowered, and catalytic activity is reduced due to aggregation and crystallization. Therefore, to improve this, it is necessary to improve the dispersion properties of the main/co-catalyst.

**Fig. 9:**
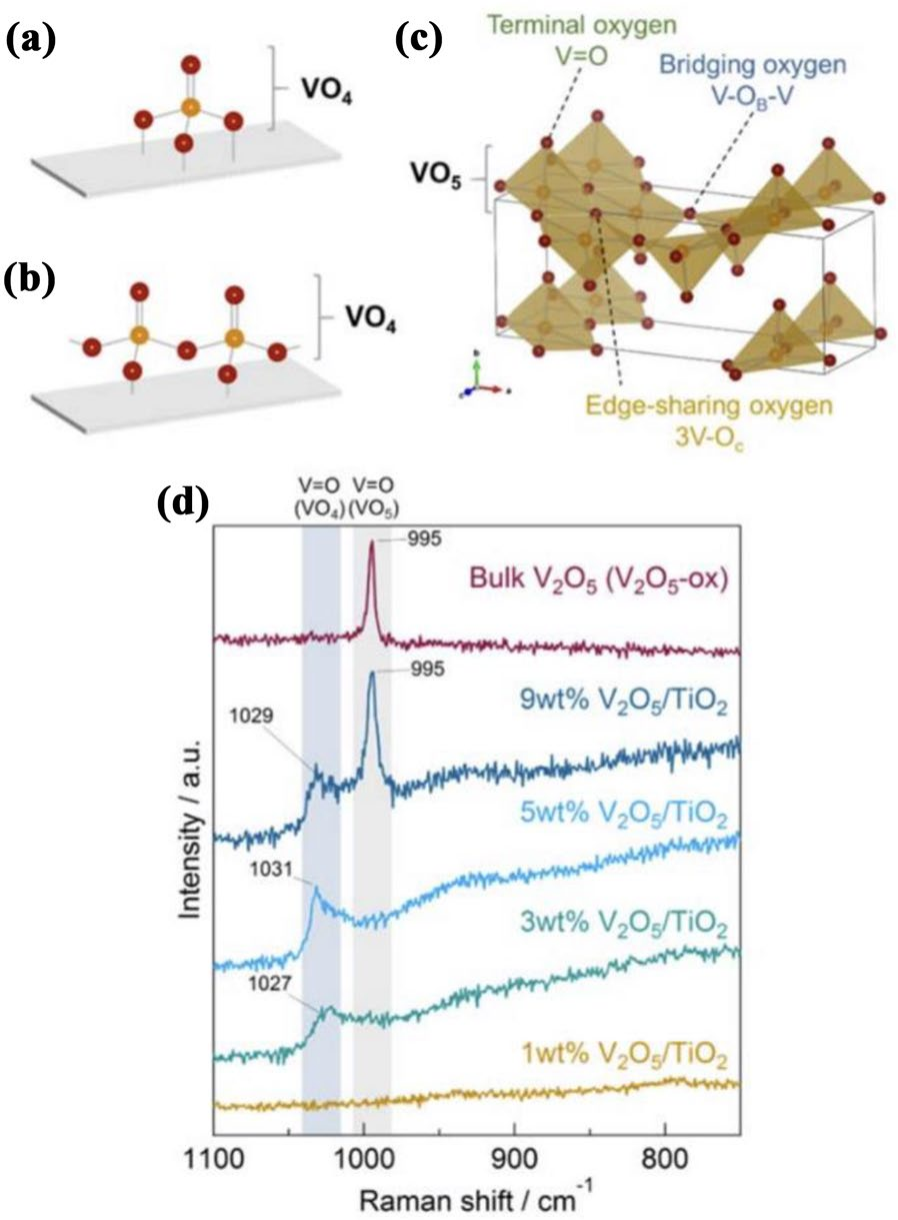
Structures of supported **a** monomeric and **b** oligomeric VOx units. **c** Crystal structure of orthorhombic phase V_2_O_5_ (Pmnm, No.59). **d** Raman spectra of bulk V_2_O_5_ (V_2_O_5_-ox) and TiO_2_-supported V_2_O_5_ catalysts (1, 3, 5 and 9 wt % V_2_O_5_/TiO_2_) [76]

### Addition of promoter and support for V-based catalyst

Table 3 lists the various compositions of vanadium-based SCR catalysts, including the main catalyst and support contents. The activity temperature window is defined as the region where more than 90% of NOx removal activity occurs for each synthesized catalyst, and specific results are summarized. Various components such as W, Mo, Ba, Ce, Mn, and Sb are added as co-catalysts to the vanadium-based SCR catalyst [44–52]. The W and Mo are the most widely used commercial co-catalysts and demonstrate thermal and structural stability and resistance to sulfur. In particular, W extends the working temperature of vanadium-based catalysts from low to high-temperature ranges [53], and although molybdenum has arsenic poisoning resistance, it exhibits a drawback of generating N_2_O at high temperatures. However, as low-temperature catalysts have recently been in the spotlight, the widespread use of Mo is increasing as a co-catalyst [54]. This interest has also led to many studies related to Mn and Ce, particularly related to the effect of Mn on NOx removal activity according to its oxidation states of + 1 to + 4. The Mn shows high NOx removal characteristics even at low temperatures (~ 150–200 °C). However, in the presence of SO_2_, MnSO_4_ is formed, and catalyst acid sites are poisoned [55]. Furthermore, Ce is widely used as a co-catalyst because of its high oxygen storage capacity and easy oxygen storage and release due to the Ce^4+^ ↔ Ce^3+^ redox shift [56–58]. Based on the oxygen vacancies present on the surface, active oxygen chemically adsorbed on the surface moves rapidly in the Ce-based catalyst and promotes the fast SCR reaction [5]. The Zr expands the catalyst operating temperature range by inhibiting particle aggregation and improving acid site dispersibility and has high thermal stability and SO_2_ resistance; however, its excessive addition promotes ammonia oxidation [59, 60]. When Sb and Nd are added in small amounts, they prevent catalyst poisoning by SO_2_ and water, and promote the decomposition of ammonium bisulfate (ABS) [61–64]. As a NOx adsorbent, Ba is used as a co-catalyst to adsorb NOx at low temperatures [26]. In addition, many researches have been conducted in which many transition metals and rare earth metals are used as co-catalysts for SCR catalysts [65–71].

**Table 3:** Comparison of different types of vanadium-based SCR catalysts

Main catalyst	Co-catalyst	Support	Content of V (wt%)	Content of co-catalyst (wt%)	Activity temperature window ( °C)	Refs.
V	–	TiO_2_	2	–	200–300	[81]
	–	TiO_2_/SiO_2_	0.1	–	420–500	[87]
	–	AC(activated carbon)	1 ~ 5	–	200 ~ 400	[82–84]
	–	CNT	2.35	–	100–250	[85]
	–	carbon-coated monoliths	5	–	120–250	[86]
	–	CeO_2_	0.75	–	250–350	[44]
	W	TiO_2_	1 ~ 5	5, 6.5, 8	200–450	[19, 45, 46]
	W	ZrO_2_	3	20	250–500	[47]
	W	CeO_2_-TiO_2_	1	10	250–475	[48]
	Mo	TiO_2_	1.5 ~ 3	1, 6	250–400	[49, 53]
	W, Mo	TiO_2_	2.5	6 ~ 9	200 ~ 450	[50, 51]
	W or Ba	TiO_2_	2	-	250–450	[52]
	W, Mo, Zr, Sn	AC(activated carbon)	5	3 ~ 5	150–250	[59]
	W, Ce	TiO_2_	0.2	5	220–400	[56]
	Ce	TiO_2_	0.5	5	300–450	[57]
	Ce	Ti-Zr	3	–	210–450	[58]
	Sb	CeO_2_-TiO_2_	2	2 ~ 10	250–450	[62, 63]
	Sb	CeO_2_	4	2	200–400	[64]
	Fe, Mn, Cu, Cr	mesoporous carbon-coated monoliths	3	1	150–200	[65]
	Fe, Co, Ni, Cu, Sr, La, and Ce	TiO_2_	1	2	250–550	[66]
	Mn, Cu, Sb, La, Mo, Ce	TiO_2_	1	3 ~ 7	300–450	[67]
	Sm, La, Ce	Al_2_O_3_	10	10	–	[68]
	La, Ce, Pr, Nd, Sm, Gd, Tb, Dy, Er	TiO_2_-WO_3_-SiO_2_(TWS)	1.9	4.6–5	250–400	[69]
Ce-V		TiO_2_-WO_3_-SiO_2_(TWS)	1.5	WO_3_ 9 SiO_2_ 10	300–500	[69]
Fe0.2Er0.8VO_4_		TiO_2_-WO_3_-SiO_2_(TWS)	8	TWS(81:9:10)	250–450	[70, 71]
CeVO_4_ nanorod	Zr	–	19	2 ~ 50	200–400	[60]

### Effect of Vanadium oxide states

As a representative transition metal, vanadium contains various valence states, such as + 2, + 3, + 4, and + 5, in the form of VO_2_, V_2_O_3_, and V_2_O_5_ [72]. Vanadium is mainly present in the form of + 4 or + 5 oxide states, but the rate at which side reactions such as SO_2_ oxidation and N_2_O generation occur and the low-temperature catalytic efficiency change, depend on the ratio of oxidation states present [73]. Therefore, studies on pH control in an aqueous vanadium precursor solution, changing the calcination temperature and maintenance time, and using various precursors are of great interest [54, 74, 75]. Previous studies have shown that the vanadium-oxalate complex undergoes thermal decomposition to V_2_O_5_ in the presence of + 4 and + 5 compounds at approximately 270 °C. Therefore, to control this, the amount of V^4+^ was changed by changing the calcination time under a temperature of 270 °C [76]. Inomata et al. found that the shorter the calcination time (at 270 °C), the higher the fraction of + 4 oxides, yielding an amorphous catalyst with a greenish color. As the calcination time increases, most vanadium exists in the + 5 form, resulting in a highly crystalline yellow catalyst. In addition, it was suggested that the NH_3_-SCR reaction rate was faster based on the high redox cycle and Lewis acid site of bulk vanadium oxide than that of V_2_O_5_ dispersed in the support. Youn et al. explored a V_2_O_3_ catalyst containing 5 wt% V, and reported that it had a high SCR efficiency across a wide temperature range and formed the least N_2_O when it existed in the + 3 oxidation state rather than in the + 4 or + 5 oxidation states, and therefore, the N_2_ selectivity was high. Additionally, when defective V_2_O_5−x_ was formed on the surface, it had a lower NH_3_-SCR reaction energy barrier than that of crystalline V_2_O_5_, and therefore this study showed that high activity vanadium catalysts could exist even at low temperatures [77].

### Effect of Vanadium surface density

Inomata et al. reported that VOx exists as monomeric and oligomeric VOx units on the surface of the support, and crystallized vanadium shows an orthorhombic phase V_2_O_5_ crystal form [76]. At a low surface concentration (< 2 V atoms/nm^2^), vanadium exists in the form of monomeric vanadyl without V–O–V bonds. As the vanadium content increases (2–8 V atoms/nm^2^), it adopts a V–O–V bridge and exists in the form of oligomeric vanadyl. At high surface densities greater than 8 V atoms/nm^2^, it exists in the form of crystallized V_2_O_5_ nanoparticles (Fig. 8) [78].

**Fig. 10:**
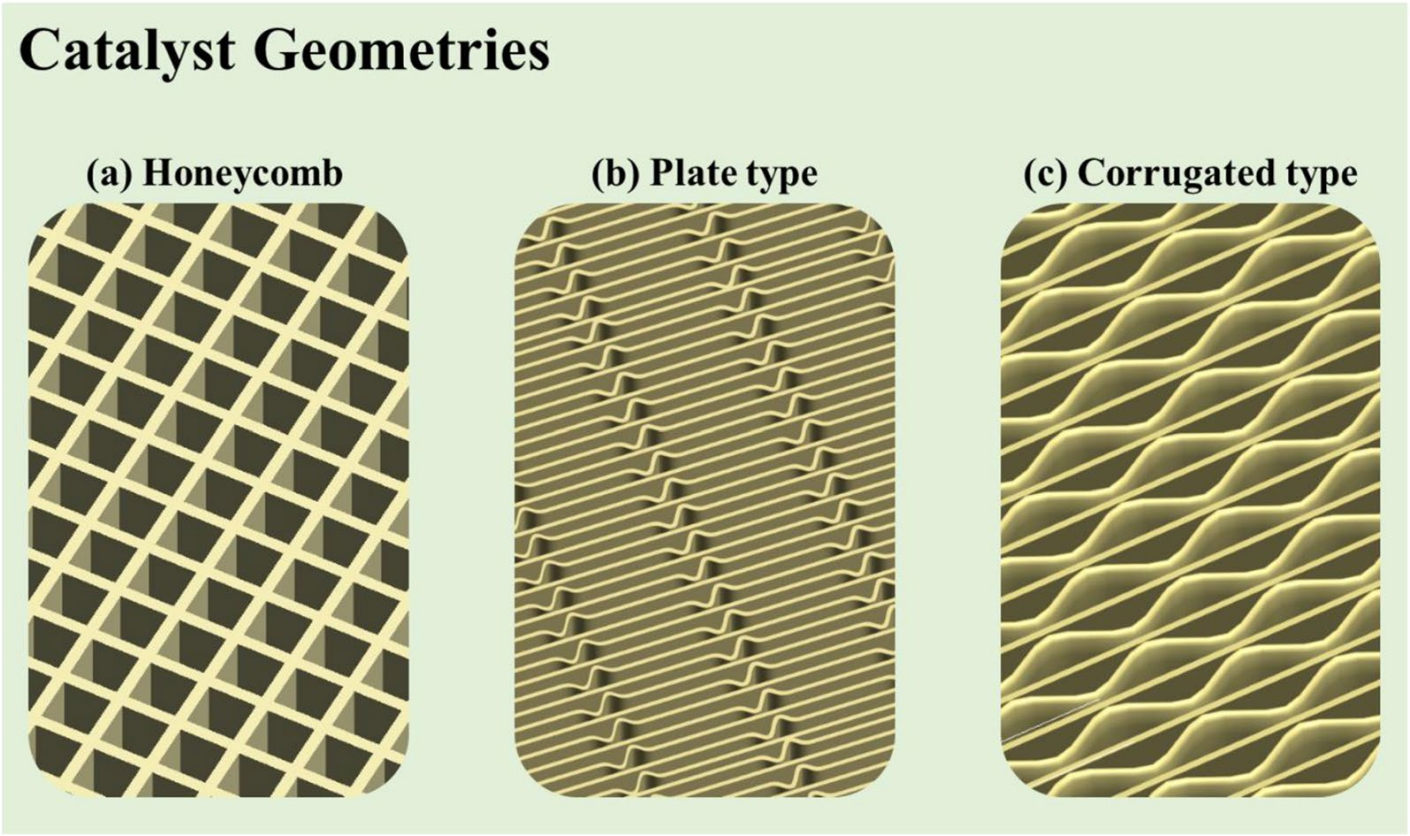
Type of monolith SCR catalysts: **a** honeycomb monolith, **b** plate, and **c** corrugated type catalyst

The binding forms of vanadium can be confirmed through Raman spectroscopy (Fig. 9), and the binding form changes depending on the vanadium content and the resulting surface density. Therefore, the catalytic efficiency also changes [76].

**Fig. 11:**
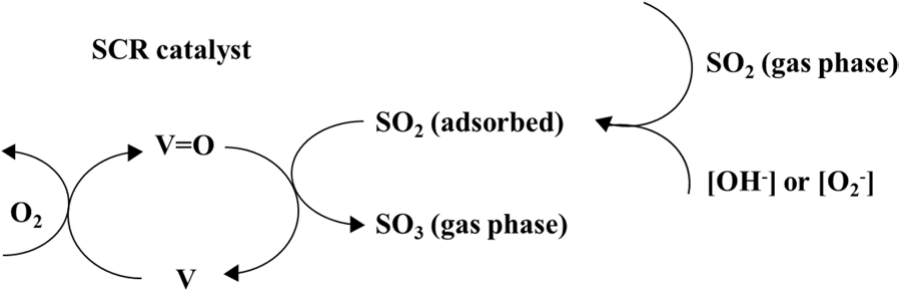
Catalytic oxidation process of SO_2_-SO_3_ under the action of V_2_O_5_

He et al. suggested that when the vanadium content is less than 1.3 wt%, 80% or more of the vanadium is monomeric [79]. When the vanadium content is 1.3–3 wt%, the monomeric fraction decreases rapidly, and the polymeric fraction increases to 33%. According to their results, polymeric vanadyl species show higher NH_3_-SCR efficiency compared to that of monomeric species owing to the shorter reaction pathway for regeneration of catalytic oxidation–reduction reaction and lower reaction energy barrier for the catalytic reaction cycle [79].

## Dispersion of active catalytic materials

Because catalysis is a surface reaction, it must be supported on a porous support. The choice of support is determined by the need for high dispersibility to increase catalyst efficiency and the need for it to have high thermal stability, specific surface area, and suitable mechanical properties for the intended application [80, 81]. Therefore, many researchers have used various dispersants to improve dispersibility [82–86]. It is important to finely disperse catalytically active materials without aggregation to enhance the catalyst acid sites, achieve a high specific surface area, and prevent sintering at high temperatures [87–89]. In addition, significant research has been conducted for improving catalyst dispersibility using supports with abundant functional groups [90] and having high efficiency and durability by dispersing single-atom catalysts on a support [91].

### Nanocomposite selection

Nanoparticles are preferentially reduced precursor materials to form small particle clusters, which can aggregate to form stable particles. Therefore, Ye et al. reported nano-dispersed catalyst materials on the surface by synthesizing the support at the particle formation stage, inhibiting the formation of large size of these particles, and thus exhibiting high NOx reduction efficiency with a smaller catalyst content [92, 93]. Using commercial reduced graphene oxide (rGO) as a support, an MnCe/rGO composite was prepared in which Mn and Ce were nano-dispersed. The catalytic efficiency was increased owing to a high specific surface area without aggregation of the active material. It was sufficiently moldable to be synthesized even with a 1-inch SCR catalyst [94]. In addition, to retain both the advantages of the high specific surface area of rGO and the abundant oxygen functional groups of GO, a catalyst using a GO-r support subjected to thermal reduction after supporting an active material on the GO surface was synthesized [8]. Next, surface-treated graphene was synthesized using N-doped graphene with nitrogen functional groups, which have higher thermal stability than oxygen groups [95]. The N-rGO support exhibited the highest dispersion and particle aggregation inhibition properties. The N-rGO has an appropriate amount of oxygen and N-functional groups on the surface, and thus demonstrates excellent thermal stability at high temperatures and efficient NOx removal characteristics at low temperatures. Surface oxygen functional groups on graphene act as anchoring sites which play an important role in preventing the aggregation of catalytically active materials and dispersing nano-sized particles as the support.

In addition to graphene, studies related to a catalyst containing vanadium and tungsten active materials dispersed using an oxygenated carbon nanotube (O-CNT) support on which an oxygen group is formed through surface acid treatment and having dispersibility and thermal stability of a TiO_2_ support with nitrogen functional group were conducted [88, 96]. In addition, a study was conducted using hexagonal boron nitride, which has a high melting point of 3000 °C and excellent thermal stability, as a support synthesized h-BN with a porous structure on the surface due to catalytic etching by the transition metal. When the active material is dispersed in this porous structure, the phase is thermally stabilized with high dispersibility, and phase changes and agglomeration are suppressed [97]. As a result, it was possible to nano-disperse the catalytically active material down to 10 nm by utilizing the surface defect anchoring sites.

### Effect of surface modification on catalytic activity (structure and morphology)

Catalytic performance is determined by the composition of active catalytic materials, variations of which could improve catalytic efficiencies. However, studies to minimize the NH_3_ slip and to develop the SCO catalyst are required owing to the current lack of researches. Therefore, it is difficult to utilize more than a specific amount of active catalytic materials, and it is naturally limited in the linear efficiencies line. To enhance the catalytic performance despite relatively lower contents of active materials, various studies have been conducted concerning surface modification such as functionalization and structure deformation. A phase transition in catalyst structures, such as hollow and kegging structures [98], is also one of the considerations. In the previous literature, active materials were easily deposited on modified and/or functionalized support, nanotubes, APT(Hydroxyapatite), controlled pores/defects, and exchanged sulfated species [99]. Deliberate surface sulfation can increase the active oxygen sites leading to the enhancement of NH_3_-chemisorption [100, 101]. Zhang et al. [102] reported on the influence of sulfation in a heterogeneous system with an iron-based catalyst. The enhancement of acid sites (Brønsted acidity and Lewis acid strength) and NH_3_-adsorption show positive effects, not only increasing efficiencies at the relatively higher temperature but also improving the resistance to SO_2_. In addition, the tolerance to H_2_O and SO_2_ is improved because SO_4_^−^ species formed by sulfation act as acid sites to increase the amount of adsorbed NH_3_. Similarly, the catalysis surface shows the mentioned effects due to modification by acidification using acids such as HCl, HNO_3_, H_3_PO_4_, and H_2_SO_4_ [103, 104]. Chenglong et al. [103] reported an improvement in the catalytic activity, NOx conversion, and N_2_ selectivity by acidification in the following order: H_2_SO_4_ > H_3_PO_4_ > HNO_3_ > HCl. In particular, surface acidity was significantly improved while increasing the Brønsted acid sites (M–O–NH_4_^+^), and it naturally yielded reducibility and surface chemical adsorption. Other studies have been conducted using H_4_PO_7_ to synthesize a nano-hollow structure, increasing surface oxygen storage capacity and acid sites [104]. In other work [105], the structure, morphology, and size of pores influenced the amount of NH_4_^+^ adsorbed for high N_2_ selectivity. In addition, a study was conducted on the role of pore diffusion in determining the active site in NH_3_-SCR reaction, and enhanced activity was investigated by controlling the more significant textile properties such as specific surface area, pore size, and structure. This work also explained that particles form the active sites, along with the correlation between the pore diffusion and actual activation energies [106]. The pore size in TiO_2_ results in a significant impact on active catalytic species [107], and the influence of meso- and micro-pores was also studied.

Consequently, it has been reported that the catalytic activity and physicochemical properties change while the vanadium bond is altered by pore size and structure effect, and the suitability of mesopores for catalytic properties is suggested. Hence, research on modification using silicon (Si) was conducted to enhance H_2_O resistance and improve catalytic activity by controlling pore textile properties [108]. The pore size and structure positively affect the dispersibility and acidity of catalytically active materials such as vanadium and tungsten caused by inhibiting and decreasing the specific surface area and improving the hydrothermal stability. The larger volume of mesopores can have more surface oxygenation groups, naturally leading to improved catalytic performance. Accordingly, research is being conducted to control pores through the use of porous materials based on carbon (C) [109, 110]. Various researchers [111] have studied carbon-based materials, and the studies have mainly focused on enhancing catalytic performance through chemical surface modification. However, the occurrence of N_2_O is also promoted, leading to the possibility for re-oxidation to NOx [112].

Activated carbon is used to modify the chemical surfaces to enhance catalytic performance. Incorporating nitrogen species into carbon enhances the catalytic activity and promotes NO_2_ formation by facilitating chemisorption of surface nitrogen species, which improves catalytic performance [113–119]. The de-NO_X_ efficiency behavior of catalysts with carbon is expected to increase due to the surface porosity. Most studies of surface functionalization have focused on adding oxygen or nitrogen via chemical or physical methods. Nitrogen functionalities can act as adsorption sites for NO_X_, and the methods are viscose-based activated carbon fibers (VACF), oxygen plasma, and nitric acid modification [120]. Among them, oxygen plasma treatment can improve and increase the oxygen functional groups on the surface, and it seems to improve pore distribution, dispersion of active catalytic material, and catalytic properties. Doping is widely used as a modification to improve catalytic activity. The S-doping of the catalyst improved its redox property, which was beneficial to the catalytic property. Zhang et al. [121] reported that sulfur could lead to more NH_3_ adsorption species through S-doping and increase the oxygen vacancy, leading to excellent activity. Royer et al. [122] reported the importance of structural and textural properties. This research explained that crystal morphologies and structures show significant differences in surface area and pore volume. Particles were highly dispersed on a hollow sphere- and monoclinic-structure among diverse types of supports including hollow sphere, star, rod, mesoporous, and crystalline- structures. Additionally, control of the TiO_2_ crystalline phase uses a cationic surfactant cetyltrimethyl ammonium bromide (CATB). In contrast, the structure and valence state of the active phase is controlled by changing the calcination temperature [123]. In conclusion, anatase TiO_2_ crystalline is more conducive for improving electron transfer.

## Commercial SCR catalyst monolith forms

As shown in Fig. 10, SCR catalysts are divided into three types [124]: honeycomb monolith, plate, and corrugated. The honeycomb type is an extruded ceramic structure that is easily regenerated, but it takes a long time for manufacture, and the catalyst is too heavy. The plate type is a metallic substrate. It has high thermal and mechanical durability, making it suitable for fly ash and gases (e.g., erosion, pressure drop) but with a low specific surface area. The corrugated type is based on a glass-fiber substrate; it has a large specific surface area and short manufacture period but poor durability. Catalysts normally use titanium oxides TiO_2_, alumina oxidesAl_2_O_3_, zeolite, and carbon as support. In general, TiO_2_ is used as a commercial catalytic support, and zeolite is used according to the condition of exhaust gases in the mobile source.

**Fig. 12:**
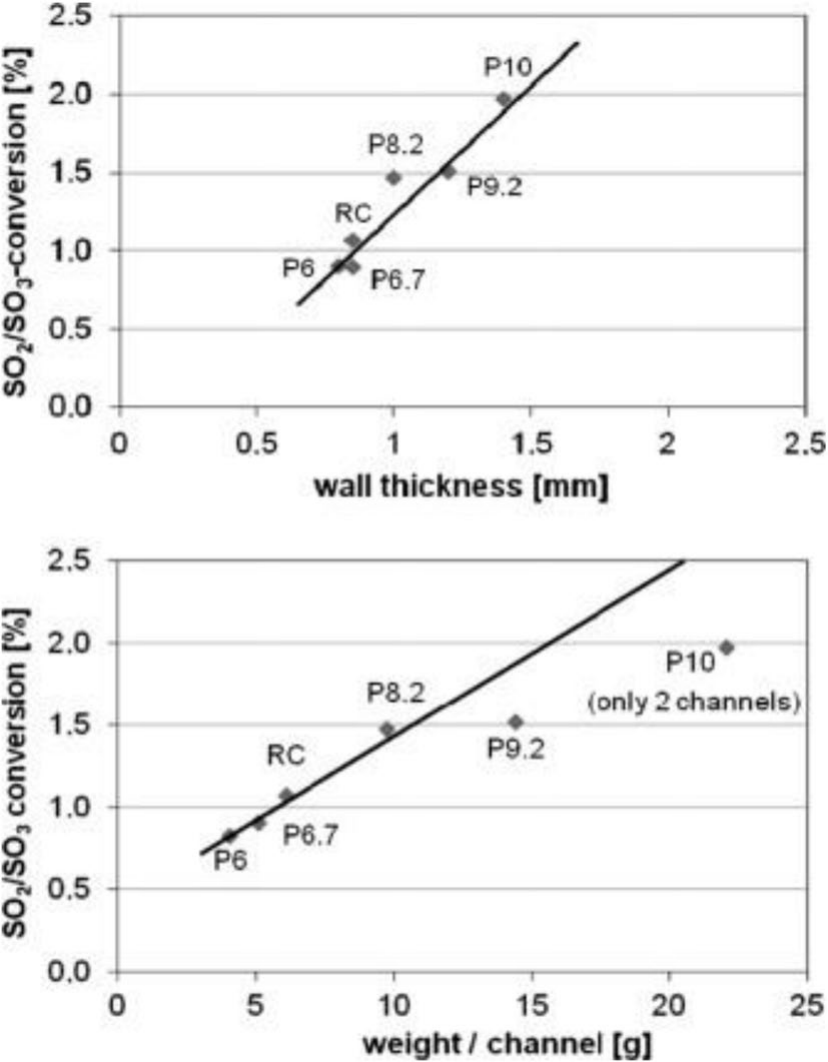
Correlations between catalyst wall thickness and channel weight and SO_2_-SO_3_ conversion [124]

### Reducing SO_2_–SO_3_ conversion and NH_3_ slip (oxidation NH_3_)

The main fuels for coal-fired electrical power plants are bituminous coal or biomass mixing coal. Therefore, reagents to sodium-based absorbents, including calcium- and magnesium-based, are used for sulfur removal. Additionally, ammonia is used as the reducing agent in SCR, but it is oxidized at high temperatures (> 380 °C). Ammonia slip in stationary sources, i.e., coal-fired and chemical power plants, causes equipment corrosion and negative side reactions. Therefore, the SCR system of stationary sources is adjusted to an NH_3_/NO_X_ ratio to alleviate ammonia slip. However, it leads to a serious problem of reduced denitration efficiency. Therefore, the commercial V–W(Mo)/Ti catalyst is used considering SO_2_–SO_3_ conversion. The SO_3_ is generated from an SCR system in a coal-fired boiler and wet electrostatic precipitator, and it reacts with ammonia, corroding the SCR system and deactivating the catalyst. Equation (2) shows that the SCR catalyst converts SO_2_ in coal-fired sulfur to SO_3_, thereby, damaging equipment when it generates sulfuric acid on reaction with water vapor present in combustion gas and forms gypsum from the CaO in fly-ash materials. Ammonium sulfate (AS) and ammonium bisulfate (ABS) are generated by the reaction of SO_3_ with excess ammonia, i.e., slipped-NH_3_.

The AS is deposited in catalyst pores and plant equipment, causing problems concerning corrosion, clogging, and performance degradation. The engineers in catalyst application and manufacturers generally require a low SO_2_–SO_3_ oxidation rate (less than 2%) [125–131]

6 $${\text{SO}}_{{2}} {-}{\text{SO}}_{{3}} {\text{conversion }}\left( \% \right) \, = \, \left( {{\text{SO}}_{{3}} {\text{outlet}}/{\text{SO}}_{{2}} {\text{inlet}}} \right) \, \times { 1}00\%$$

Vanadium provides the active site of the SCR reaction, but it also causes oxidation of SO_2_ to SO_3_. Therefore, many researchers have tried to control the SO_2_–SO_3_ conversion, such as the use of low sulfur coal and absorbents (magnesium, calcium), as well as improving the desulfurization system. In addition, attempts have been made to suppress this reaction by changing the composition of the commercial SCR catalysts and modifying the surface.

**Fig. 13:**
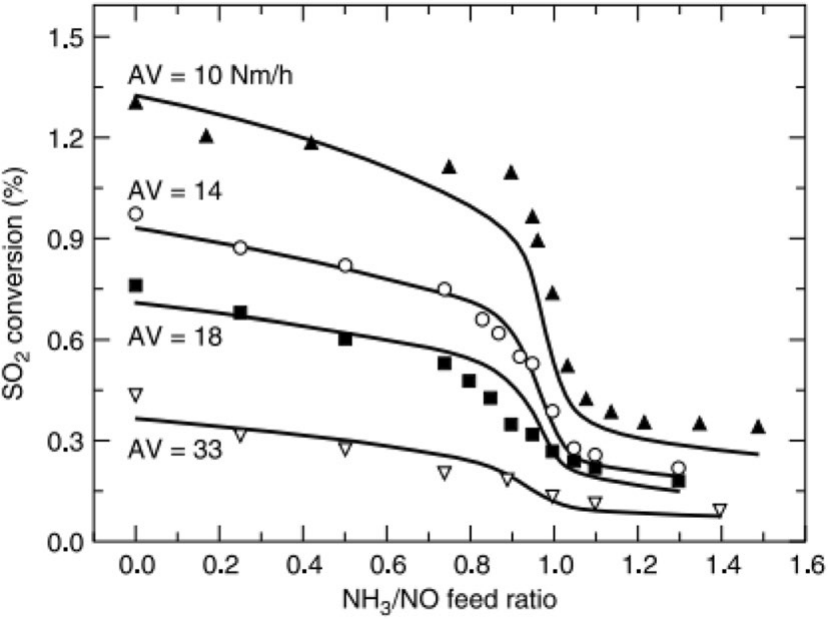
Effect of NH_3_/NOx ratio and AV to SO_2_-SO_3_ conversion [134]

The SCR reaction occurs on the surface, whereas SO_2_–SO_3_ conversion is a diffusion-based reaction (see Fig. 11). The variables that affect SO_2_–SO_3_ conversion are catalyst composition, temperature [132], catalyst geometry (pitch, open area, wall thickness) (see Fig. 12) [124, 133–136], gas composition, and operating conditions (see Fig. 13). Therefore, improved durability can be obtained through the inhibition of oxidation on the surface by preventing SO_2_-adsorption. The results indicated a linear dependence on catalyst wall thickness and channel [124, 128, 135]. For this reason, corrugated type catalysts show lower SO_2_–SO_3_ conversion and are better in this respect than honeycomb and plate-type catalysts. The SO_2_–SO_3_ conversion rate indicated a difference with respect to the velocity of the gas passing through the catalyst, and the result indicated easier diffusion, as mentioned above, yielding lower AV [134].

However, as the rationale for catalyst choice cannot be limited to a simple SO_2_–SO_3_ conversion property, the optimal catalyst monolith is determined by the exhaust gas conditions for each site. Consequently, further research is required for reducing the SO_2_–SO_3_ conversion rate.

**Fig. 14:**
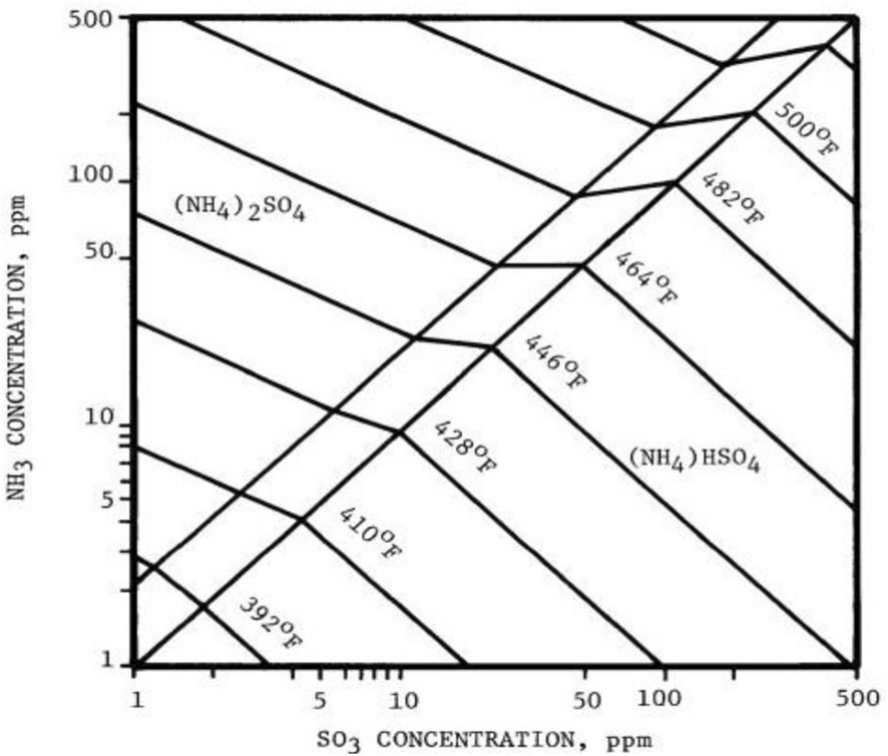
Influence of NH_3_ concentration on SO_3_ formation

Studies on the SO_2_–SO_3_ conversion by Saltsburg et al. [137] and Morikawa et al. [138] has been in progress since 1979. Scheffknecht et al. [126] and Beretta et al. [139] studied the influence of each factor mentioned above. Scheffknecht et al. [126] reported the effects of SO_2_–SO_3_ conversion on catalyst components (Cu, V, W), the concentration of SO_2_ and H_2_O, and flue gas velocity. The SO_2_–SO_3_ conversion increased with increasing SO_2_ concentration [140]. However, a non-linear trend was observed from the saturation point of SO_2_–SO_3_ conversion [140]. The SO_2_–SO_3_ conversion can also be explained by the Arrhenius and Eyring equations. These equations show that the conversion rate increases with increasing temperature. However, the H_2_O range is not dependent on the SO_2_–SO_3_ conversion reaction. This finding was contradicted by other reports, which have found that the addition of H_2_O influenced the generation of SO_3_ by inhibiting SO_3_ adsorption [128, 129]. Along with influenced factors in the SCR process of SO_2_–SO_3_ conversion, Yang et al. [133] evaluated and reported various compositions in flue gases, such as O_2_, NH_3_, NOx, SOx, H_2_O, and CO_2_ [128, 129], as well as catalyst components TiO_2_, V_2_O_5_, WO_3_, Al_2_O_3_, BaO, and SiO_2_. According to their report, the gas composition is directly influenced by SO_2_–SO_3_ conversion. The NH_3_ especially affects the SO_2_–SO_3_ conversion rate, and when the concentration of NH_3_ exceeds the NO content, it continues to decrease the SO_2_/SO_3_ conversion rate. Wang et al. explained that the presence of NH_3_ inhibits the formation of SO_3_, while the oxidation of SO_2_–SO_3_ conversion can be controlled by readily reacting surface-oxygen with NH_3_ [141]. The concentration of O_2_ in the exhaust gas is not impacted by SO_2_–SO_3_ conversion [130]. Furthermore, vanadium content directly influences SO_2_–SO_3_ conversion, and vanadium surface coverage and density are related to SO_3_ oxidation [130, 142]. That is to say, SO_2_–SO_3_ conversion is connected to only one surface vanadate site. Commercial catalysts use the V–W/Ti components, i.e., a vanadium-based catalyst with a co-catalyst to improve the SO_2_–SO_3_ conversion. Thus, many studies have been conducted on SO_2_–SO_3_ conversion, and the main factors included the suppression of SO_3_ through the use of a co-catalyst and optimized support. In general, vanadium-based catalysts are commercially represented using tungsten oxide (WO_3_) and molybdenum oxide (MoO_3_) as promoter. Many studies have shown that WO_3_ inhibits SO_2_–SO_3_ oxidation, including the report by Ismagilov et al. [143], and the rate is affected by the tungsten content [93, 143]. However, based on other reports, a consensus has not yet been reached on the influence of tungsten content on the SO_2_–SO_3_ conversion rate [133, 142]. Although molybdenum has the desirable property of being resistant to SO_2_ by naturally restricting the SO_2_–SO_3_ conversion rate, other catalysts have been used more widely in the industry than molybdenum [144]. However even with this composition, because the SO_2_–SO_3_ conversion rate is limited, studies are being conducted on the effects of metallic bonds and diversification of catalytic composition by incorporating rare earth and transition metals (Cu, Ba, Mg, Fe, Ge, Zn, Ta, and Y). Wachs et al. [130, 131] studied the turnover frequency of SO_2_ oxidation by co-catalysts on TiO_2_ supports. The oxidation rate was observed in the order of: V_2_O_5_ > Fe_2_O_3_ > Re_2_O_7_ > CrO_3_ > Nb_2_O_5_ > MoO_3_ > WO_3_ > K_2_O. The effect of iron (Fe_2_O_3_) on SO_2_–SO conversion has been researched by Wang et al. [145]. With an increase in the Fe_2_O_3_ loading, the SO_2_–SO_3_ conversion also increased. However, studies have reported that excessive iron loading causes a non-uniform distribution of active components on the catalytic surface and an increased SO_2_–SO_3_ conversion [145]. Also, to those mentioned in other reports, the addition of barium (Ba) as a co-catalyst showed low SO_2_–SO_3_ conversion [143]. The Ba species are an important factor in the SO_2_–SO3 conversion rate, affecting surface acidity. Ceria (Ce) has sulfur resistance and inhibits SO_2_–SO_3_ oxidation; therefore, it is generally used for Mn-based catalysts vulnerable to sulfur [146]. The rare earth niobium (Nb) showed the greatest resistance to SO_2_ and inhibited surface reaction to SO_2_. Therefore, using Nb, a decrease in SO_2_ oxidation at temperatures below 350 °C was demonstrated [93, 130, 147]. Germanium (Ge) and Zinc (Zn) components are effective promoters for retarding the SO_2_–SO_3_ conversion [142]. In addition, research on Cu-containing catalysts has shown that they have the disadvantage of increasing the SO_2_–SO_3_ conversion; however, they have the advantage of improving the mercury oxidation rate [122]. Xiang et al. [129] have studied the SO_3_ generation, and Yang et al. [133] focused on the factors of SO_2_–SO_3_ conversion. Furthermore, Hitachi-Zosen reported that SO_3_ reacted with NH_3_ generated from ABS and AS, as shown in Fig. 14.

The NH_3_ oxidation generates NO and N_2_O, and therefore, it must be suppressed. The NH_3_ slip is generally adjusted to 2 ppm or less [148]. The basic method for inhibiting NH_3_ slip is to reduce the NH_3_/NO ratio. However, in this case, it is necessarily directly related to the decrease in the de-NOx efficiency, and therefore, unfortunately, its application is difficult. For this reason, research has been conducted with a focus on the selective catalytic oxidation (SCO) technology and removal of residual NH_3_ after reaction. The use of co-catalysts to inhibit NH_3_ oxidation is also related to slip prediction models. However, NH_3_ can also be oxidized by major substances in the catalyst, such as vanadium, tungsten, and molybdenum; therefore, this factor should also be considered [148, 149]. As the NH_3_ oxidation reaction is connected to a decrease in N_2_ selectivity and inhibited NH_3_-SCR reaction, it causes a decrease in the catalyst activity [150]. The NH_3_ oxidation reaction proceeds via the following reaction pathway.

7 $${\text{4NH}}_{{3}} + {\text{ 4NO }} + {\text{ O}}_{{2}} \to {\text{4N}}_{{2}} + {\text{ 6H}}_{{2}} {\text{O }}\left( {\text{direct oxidation}} \right)$$

8 $${\text{NH}}_{{3}} + { 5}/{\text{4O}}_{{2}} \to {\text{NO }} + {3}/{\text{2 H}}_{{2}} {\text{O}}$$

9 $${\text{2NH}}_{{3}} + {\text{ 2O}}_{{2}} \to {\text{N}}_{{2}} {\text{O }} + {\text{ 3H}}_{{2}} {\text{O}}$$

**Fig. 15:**
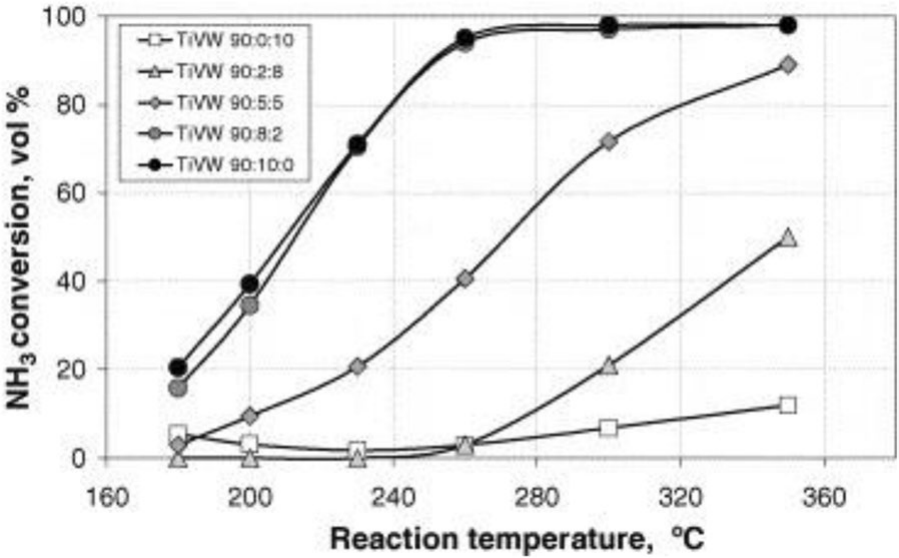
N_2_ selectivity to **a** N_2_ and **b** N_2_O during NH_3_ oxidation as a function of reaction temperature [153]

**Fig. 16:**
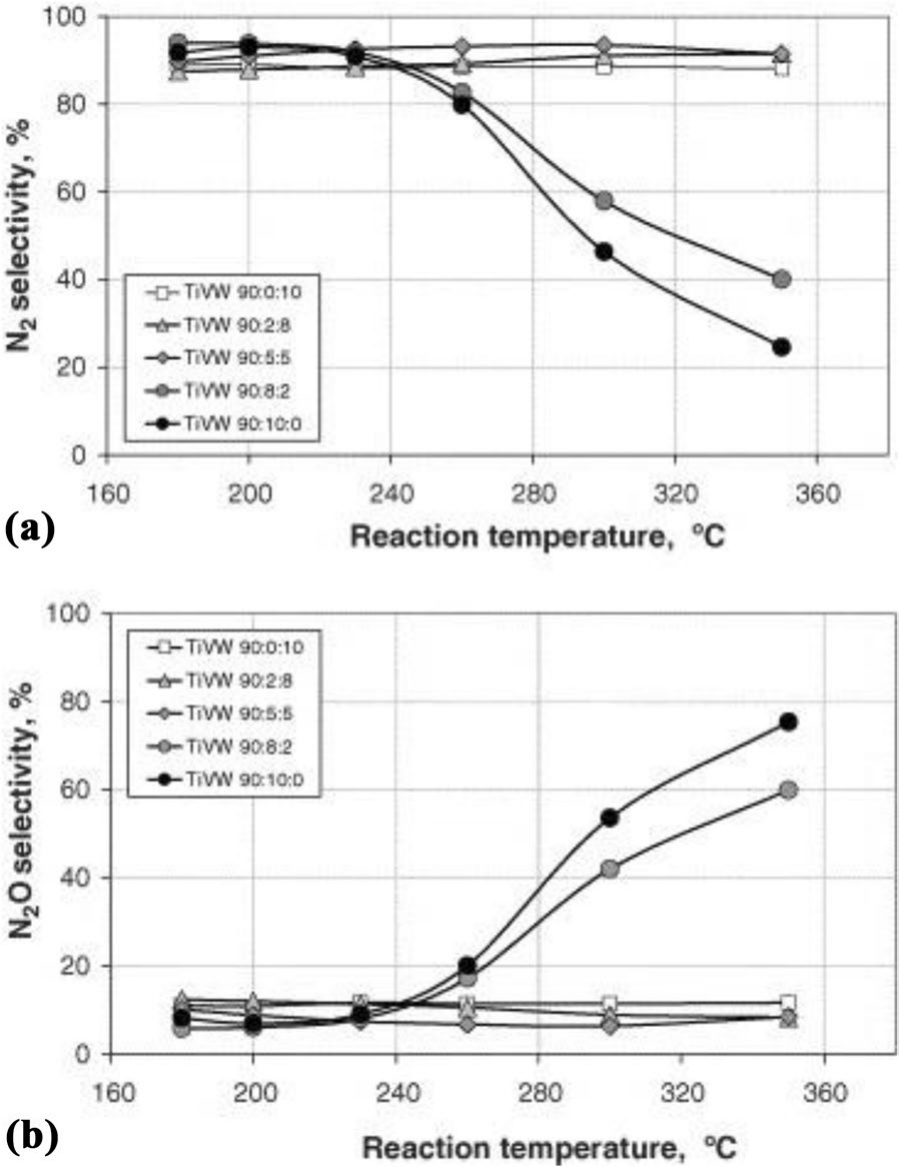
Reaction temperature effect of NH3 conversion, operating condition: space velocity at normal conditions (GHSV), 15,000 h^−1^; linear velocity, 0.1 ms^−1^; pressure, 0.1 MPa. Feed composition: [NH3] = 500 ppm; [O2] = 3 vol%; [Ar] = balance [153]

**Fig. 17:**
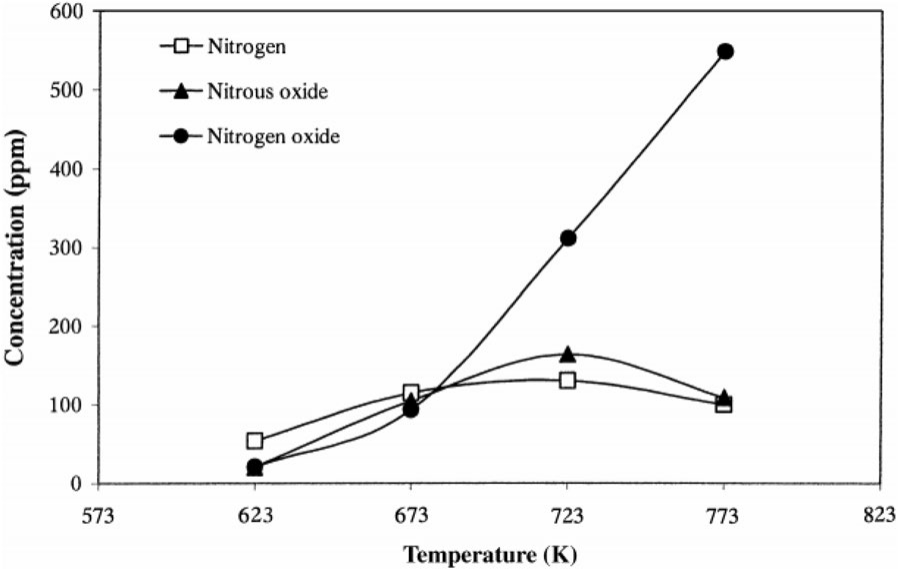
NH_3_ oxidation on vanadium catalyst [154]

In the case of an indirect reaction from NH_3_ to N_2_ and H_2_O, as shown in Eq. 7–9, there is a problem related to the generation of by-products, such as NO and N_2_O. Therefore, such a reaction must be suppressed to prevent activity degradation, as discussed above [151]. Epling et al. [152] and Blanco [153] have investigated the presence of an indirect path or direct oxidation to N_2_ after the formation of NO [152] or N_2_O (see Fig. 15) [153] during oxidation from NH_3_. Indeed, to inhibit the NH_3_-oxidation, many studies have been conducted on mechanisms concerning oxidation and adsorption to examine the doping effects of W [121, 126, 133], Cu [113, 114], Ce [113, 120], Fe [113, 120], and Ru [122, 127]. The studies confirmed the kinetic scheme of NH_3_/NOx ratio, temperature, vanadium loading influence (see Fig. 16), and NH_3_-slip. In fact, the direct factors include the intrinsic rate of NH_3_ oxidation and the volume of catalyst.. In fact, the direct factors depend on the intrinsic rate of NH_3_ oxidation and the catalyst volume. Additionally, the catalyst monolith thickness and diffusion of NH_3_ were studied. In other works reported the NH_3_ oxidation depended on N_2_ selectivity. According to the research on NH_3_ reaction, ammonia oxidation increases with an increase in the reaction temperature and vanadium content, and the N_2_ selectivity decreases rapidly. Grange et al. [154] reported the relationship between NH_3_ oxidation and SCR catalyst through the DRIFT study and confirmed the connection with the V = O octagon band in DRIFTS, particularly the temperature of the SCR reaction, the de-NO_X_ efficiency, and oxidation in the vanadium-based catalyst (see Fig. 17). Furthermore, Wang et al. [155] reported on the relations between ammonia storage and slip and gas hourly space velocity (GHSV) and temperature, among other factors. This research indicated that GHSV affects NH_3_ slip by affecting the temporary NH_3_ reaction and the heating rate of the catalyst. Epling et al. [152] reported that Cu demonstrates the advantage of easier control of NH_3_ oxidation than Fe as the storage capacity for NH_3_ and NOx was higher than that of the Fe catalyst. In the V–Mo-based catalyst using Ru [156, 157], Ru inhibits NH_3_ slip at 350 °C by increasing NH_3_ decomposition efficiency and improves N_2_ selectivity to 97%. Additionally, depending on the presence of SO_2_, NH_3_ oxidation was partially inhibited. Moreover, a study on NH_3_ oxidation inhibition was conducted by doping with CaO, a catalyst poison. This research indicated that the addition of CaO improves NO formation by NH_3_ oxidation and inhibits N_2_O formation, thereby changing the NH_3_ reaction pathway. As a result, doping with CaO improves the de-NO_X_ efficiency and N_2_ selectivity [150]. However, as CaO is generally considered to be a poison substance in catalysts, more complementary research is necessary. In summary, it is essential to control the vanadium content and the amount of NH_3_ adsorbed [158]. Additionally, it is important to consider the influence of exhaust gas and flow rate [155], such as NH_3_/NOx for inhibiting the NH_3_ oxidation. However, it will be necessary that the ongoing researches focus on minimizing the NH_3_ slip and developing the SCO catalyst because of the lack of related research at present.

## Conclusions and outlook

Vanadium-based catalysts with W or Mo co-catalysts and TiO_2_ supports have been used as commercial NH_3_-SCR catalysts without any major changes to the composition for many years, despite research into new compositions. However, air quality standards are being strengthened worldwide, which has renewed interest in the development of SCR catalysts with high NO_*x*_ reduction efficiencies and excellent poisoning resistance at low operating temperatures. This review discusses the current status of research on commercial vanadium-based SCR catalysts used to reduce NO_*x*_ emissions from stationary sources worldwide.

The diverse operating conditions of SCR catalysts in actual industrial sites necessitates the tailoring of catalyst properties for a given application. For example, catalysts with physicochemical poisoning resistance to different poisoning substances (mercury, alkali metals, particulate matter, sulfur, etc.) over a wide range of operating temperatures are required; therefore, several methods have been developed to design suitable catalysts based on the intended application area. In addition, there are several methods for increasing the efficiency of SCR catalysts, including the use of different co-catalysts (W, Mo, Ba, Ce, Sb, Fe, Mn, etc.), supports (TiO_2_, Al_2_O_3_, CeO_2_, activated carbon, etc.), and vanadium species (polymeric, monomeric, and crystalline V_2_O_5_); tailoring the surface density, support application (carbon based materials, two-dimensional materials), and particle structure; and surface modification. In addition, although much research has been conducted on powder-type catalysts, this review discussed several other monolith catalysts (honeycomb-type, plate, corrugated, etc.) that may help to meet the commercial catalyst requirements for a given application. Catalyst monolith forms can be optimized for the specific operating and exhaust conditions of a given application; therefore, different catalyst morphologies are used depending on the place of use. We discussed the correlation among the reaction, geometry, and morphology of monolith catalysts to improve their performance and properties under variable operating conditions.

Environmental regulations are set to become continually stricter in the future. Therefore, improved methods of controlling the amount of N_2_O and unburned NH_3_ emitted by SCR side reactions will be required. Currently, as fossil fuels are used in industrial plants, NH_3_, an unreacted reducing agent, is mainly emitted along with NO_*x*_, CO, VOCs, UHC, and particulate matter. Therefore, there is a need to develop oxidation–reduction catalysts or module systems that are capable of reducing these pollutants simultaneously in the SCR facility. In addition, as more governments and enterprises seek to achieve carbon neutrality, new combustion technologies based on low-carbon or non-carbon fuels such as ammonia or hydrogen with conventional LNG are being applied to replace fossil fuels. These new fuels change the composition of the flue gas; however, thermal NO_*x*_ is still emitted in large quantities from combustion reactions. Consequently, continuous research is needed to ensure SCR catalyst technologies keep up with the changing field of fuel combustion. Importantly, under co-firing conditions, pollutants such as CO and unreacted NH_3_ may be generated due to incomplete combustion. Therefore, in the future, it will be necessary to not only improve the performance of SCR catalysts, but also to keep pace with new developments by designing functional reduction catalysts that can simultaneously reduce various pollutants together with NO_*x*_.

## Data Availability

Not applicable.
